# Syphilis Hepatitis Presenting as a Mimic of Primary Biliary Cholangitis

**DOI:** 10.14309/crj.0000000000000497

**Published:** 2020-12-08

**Authors:** Cody Kern, Ahmed Elmoursi, Caroline Blake, Andrew Hoellein

**Affiliations:** 1 Department of Internal Medicine, University of Kentucky Medical Center, Lexington, KY; 2 Department of Digestive Disease and Nutrition, University of Kentucky Medical Center, Lexington, KY; 3 University of Kentucky College of Medicine, Lexington, KY

## Abstract

Syphilis hepatitis is a rare cause of acute liver injury. Primary biliary cholangitis (PBC) is a progressive autoimmune disease characterized by the typical presentation of a cholestatic liver injury and the presence of antimitochondrial antibodies (AMAs). We present a case of syphilis hepatitis that presented as a mimic to PBC with positive AMA. The eradication of syphilis led to the resolution of the liver injury and down trending of the antibody level. We recommend excluding syphilis in patients with high-risk behaviors presenting with a cholestatic liver injury and positive AMA before the diagnosis of PBC.

## INTRODUCTION

Primary biliary cholangitis (PBC) is a progressive cholestatic liver disease defined by autoimmune destruction of intrahepatic bile ducts.^1^ PBC most commonly affects middle-aged women and can progress to end-stage liver disease.^2^ The typical presentation is a cholestatic liver injury pattern, marked by elevated alkaline phosphatase (ALP), gamma-glutamyltransferase, and total bilirubin. Aspartate aminotransferase (AST) and alanine aminotransferase (ALT) levels are commonly normal or only moderately elevated.^3^ Antimitochondrial antibody (AMA) is present in 95% of PBC diagnoses. Diagnostic criteria for PBC are met with the combination of an elevated AMA and ALP greater than 1.5 times the upper limit of normal.^1^

Syphilis is a sexually transmitted infectious disease caused by the spirochete, *Treponema pallidum*.^4^ The highest risk populations are men who have sex with men and persons living with human immunodeficiency virus.^5^ The staged progression of syphilis is well-documented. Primary syphilis manifests with a genital chancre, while secondary syphilis is characterized by the typical rash, fever, and adenopathy.^6^ Syphilis hepatitis is a variation that is sparingly seen in secondary syphilis; although rare, it is more common in persons living with human immunodeficiency virus (PLWH).^5^ We present a case of syphilis hepatitis that initially presented as a mimic of PBC, with a cholestatic liver injury and a positive anti-M2 AMA level.

## CASE REPORT

A 54-year-old man with medical history of human immunodeficiency virus, hypertension, hyperlipidemia, and diabetes mellitus with a previous cholecystectomy presented to the emergency department with 1 week of diffuse abdominal pain and vomiting. On arrival, the patient was afebrile and hypertensive. Additional signs and symptoms at the time of initial presentation included jaundice, scleral icterus, and a diffuse nonpruritic erythematous, macular rash located on his torso, back, and extremities, including palms. The rash emerged 3–4 days before presentation. Physical examination was negative for ascites, asterixis, hepatosplenomegaly, lymphadenopathy, or genital lesions. Social history was pertinent for unprotected sex 6 months before presentation. He denied recent travel or use of herbal supplements. Laboratory results showed K 5.6 mmol/L, Na 133 mmol/L, Cr 1.56 mg/dL, AST 91 IU/L, ALT 120 IU/L, ALP 832 IU/L, total bilirubin 6.4 mg/dL, conjugated bilirubin 4.9 mg/dL, and CD4 count 336. Viral hepatitis panel was negative. Hemogram did not show leukocytosis or thrombocytopenia. Through chart review, the patient did not have any abnormal liver enzymes in the previous 5 years. Computed topography performed showed no acute abdominal abnormalities, no evidence of biliary obstruction. To further evaluate liver parenchyma and the biliary system, a right upper quadrant ultrasound with Doppler was ordered. It demonstrated a heterogenous, coarse liver with patent vascular, no splenomegaly, and an absent gallbladder. Common bile duct measured 2–3 mm. The patient's acute kidney injury resolved with intravenous fluids, but the acute cholestatic liver injury persisted without evidence of worsening hepatic dysfunction. At this point, the liver injury was felt to be secondary to his antiretroviral therapy regimen. Dolutegravir, abacavir, and lamivudine were discontinued, and he was started on the combination regimen bictegravir, emtricitabine, and tenofovir. He was discharged home with plans for follow-up with primary care provider in 1 week.

He presented 1 week later to his primary care provider with continuous abdominal pain, jaundice, and diffuse rash. Laboratory results showed persistent cholestatic liver injury AST 92 IU/L, ALT 144 IU/L, ALP 754 IU/L, and total bilirubin 6.7 mg/dL. Further testing obtained at this time was significant for a positive syphilis immunoglobulin (Ig)G and rapid plasma regain of 1:256 and an anti-M2 AMA IgG of 83.5 U (positive >25 U) which was suspicious for syphilis hepatitis vs underlying PBC. As he had an allergy to penicillin, he was started on doxycycline for 2 weeks for the treatment of secondary syphilis. He completed the antibiotic course with resolution of abdominal pain, jaundice, and rash. Repeat laboratory work obtained 2 months later revealed normalized liver function. Once the rapid plasma regain titer returned negative, a repeat AMA IgG was tested and negative at 16.4U. Liver biopsy was not pursued because of resolution of the liver injury. Normalization of both AMA IgG level and liver enzymes after eradication of syphilis prompted the diagnosis of syphilis hepatitis associated with a false-positive AMA IgG level.

## DISCUSSION

The incidence of syphilis hepatitis is variable, particularly in PLWH. One retrospective analysis by Crum-Cianflone et al of 32 PLWH with early syphilis found hepatic involvement to be 38%.^6^ A second retrospective study found a lower rate of syphilis hepatitis in PLWH 5/50 (10%); however, it was hypothesized that there may have been an underestimation because of exclusion of patients with previous underlying liver conditions.^7^ The diagnostic criteria for syphilis hepatitis established by Mullick et al: (i) abnormal liver enzyme levels; (ii) serologic evidence for syphilis; (iii) exclusion of other causes of liver diseases; and (iv) liver enzyme levels returning to normal after appropriate antimicrobial treatment.^8^

Clinical manifestations of syphilitic hepatitis are often nonspecific. The most common signs are jaundice and a nonpruritic rash with multiple erythematous and nonconfluent maculopapular lesions, concentrated on the trunk, palms, and soles.^4,9^ Serum liver transaminases are typically mildly increased; however, the most common abnormality is a disproportionately elevated serum ALP.^8,10^ This case similarly presented with the characteristic rash and significantly elevated ALP compared with other hepatic enzymes. The highlight of this case is that the patient had an elevated anti-M2 AMA titer that is normally associated with PBC, thus complicating the initial diagnosis. Miyakawa et al previously demonstrated that false-positive reactions to the PBC-specific anti-M2 AMA reactions could occur with hepatitis A, syphilis, and rheumatoid arthritis.^11^ This study showed a return to a negative AMA titer after recovery from hepatitis A. However, such data after treatment for syphilis was not reported. Similarly, syphilis has previously been described to cause an elevation in other nonspecific mitochondrial antibodies (particularly anti-M1 AMA) but not anti-M2 AMA.^12,13^

Although our patient initially met criteria for the diagnosis of PBC, it is now apparent that the AMA was elevated because of an active syphilis infection. This is further supported by the resolution of liver injury in this patient after treatment with doxycycline. This case demonstrates the ability of syphilis to cause positive anti-M2 AMA that are typically associated with the diagnosis of PBC. As syphilis is an easily targeted cause of liver injury, we recommend testing for syphilis in patients with high-risk behaviors that present with a cholestatic liver injury and positive AMA before the diagnosis of PBC.

## DISCLOSURES

Author contributions: C. Kern, A. Elmoursi, and C. Blake wrote the manuscript, revised the manuscript for intellectual content, and approved the final manuscript. A. Hoellein revised the manuscript for intellectual content, approved the final manuscript, and is the article guarantor.

Financial disclosure: None to report.

Previous presentation: This case was presented at the American College of Gastroenterology Annual Scientific Meeting; October 25–30, 2019; San Antonio, Texas.

Informed consent was obtained for this case report.
